# Supplementary material for: Exploration of Mediators Associated with Myocardial Remodelling in Feline Hypertrophic Cardiomyopathy
Source: Animals (Basel). 2023 Jun 26;13(13):2112. doi: 10.3390/ani13132112 (PMC10339868; doi:10.3390/ani13132112)
Supplement: Supplementary file 1 [file animals-13-02112-s001.zip › Supplementary Figures S1 S2 S3 S4.pdf]

Figure S1. Immunofluorescence of LOX and lumican

A

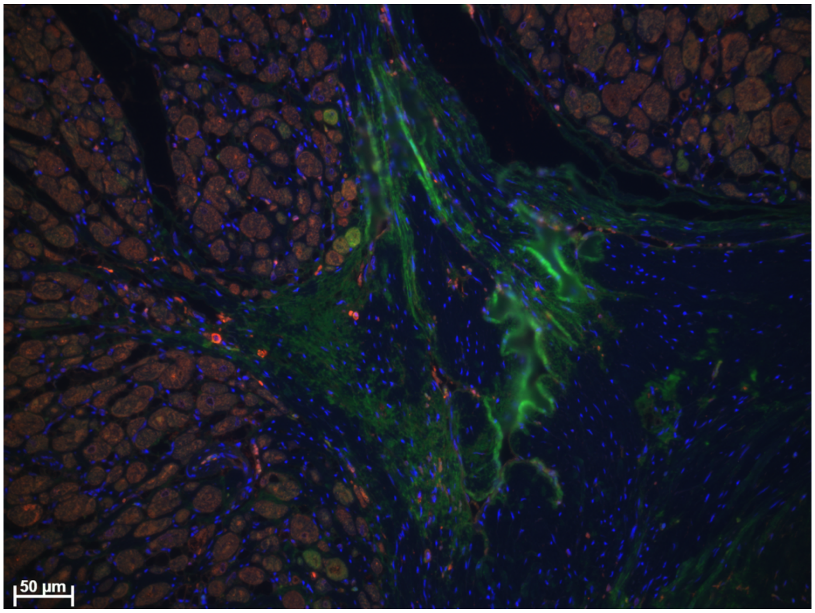

B

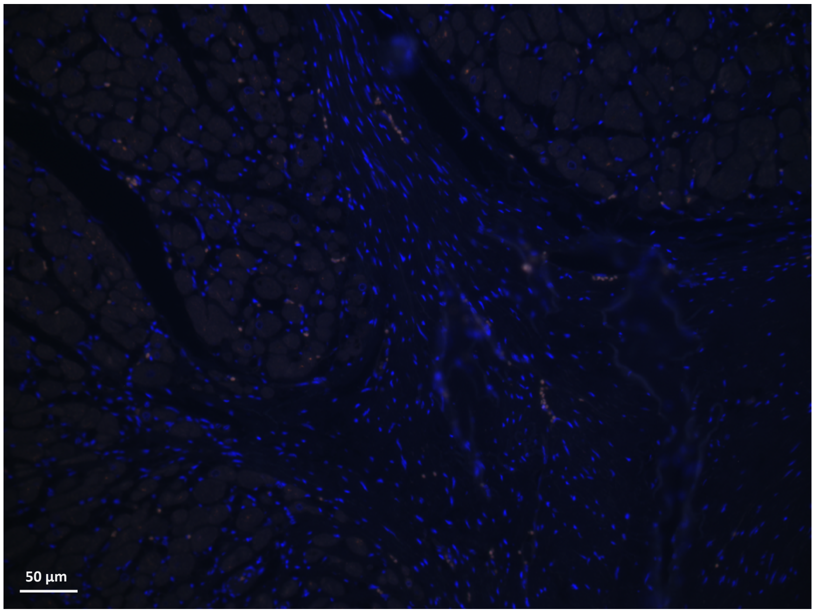

Figure S1. Immunofluorescence of LOX and lumican

(A) Feline myocardium immunostained for (1) LOX (sc-373995, or F-8, from Santa Cruz) in red, a monoclonal antibody raised in mice which is a different antibody from the one used in main text, (2) lumican in green, and (3) DAPI in blue. Image showed LOX was detected in cardiomyocytes and interstitial cells, similar to the image immunostained with the LOX antibody used in the main text which is a polyclonal antibody raised in rabbit. Image also showed strands of lumican (although not in focus) and areas in the interstitium labelled with lumican, and some cardiomyocytes labelled with lumican. (B) A reagent control for (A) by omitting the primary antibodies for LOX and lumican.

Figure S2. The uncropped images of Western Blot Analyses of TGF-β1 and TGF-β2

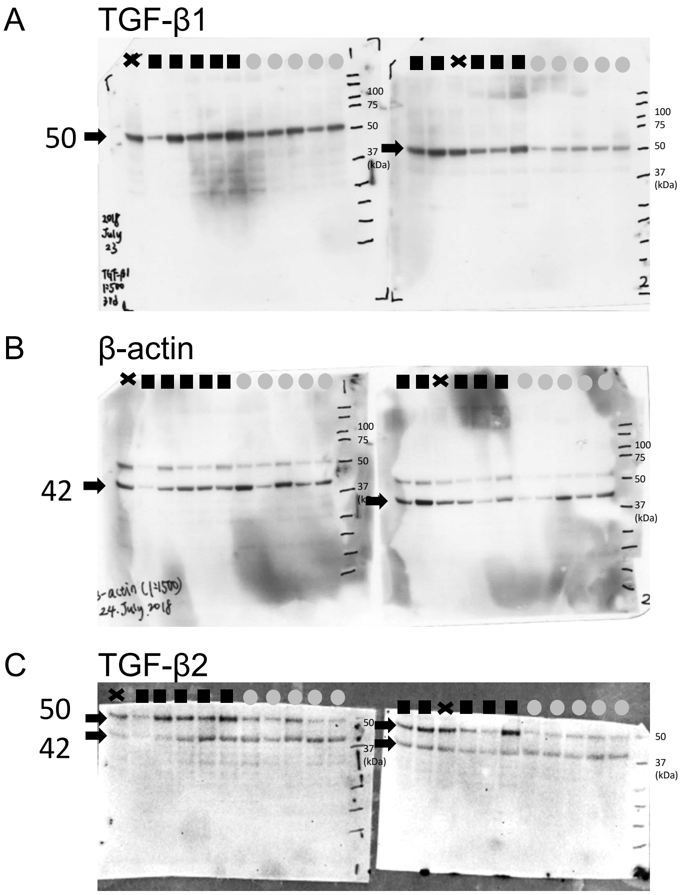

Figure S2. The uncropped images of Western Blot Analyses

(A-C) The original western blots of Figure 6A, 6D. *(Control – circle; HCM – square)*

Figure S3. Exploration of soluble and insoluble collagen

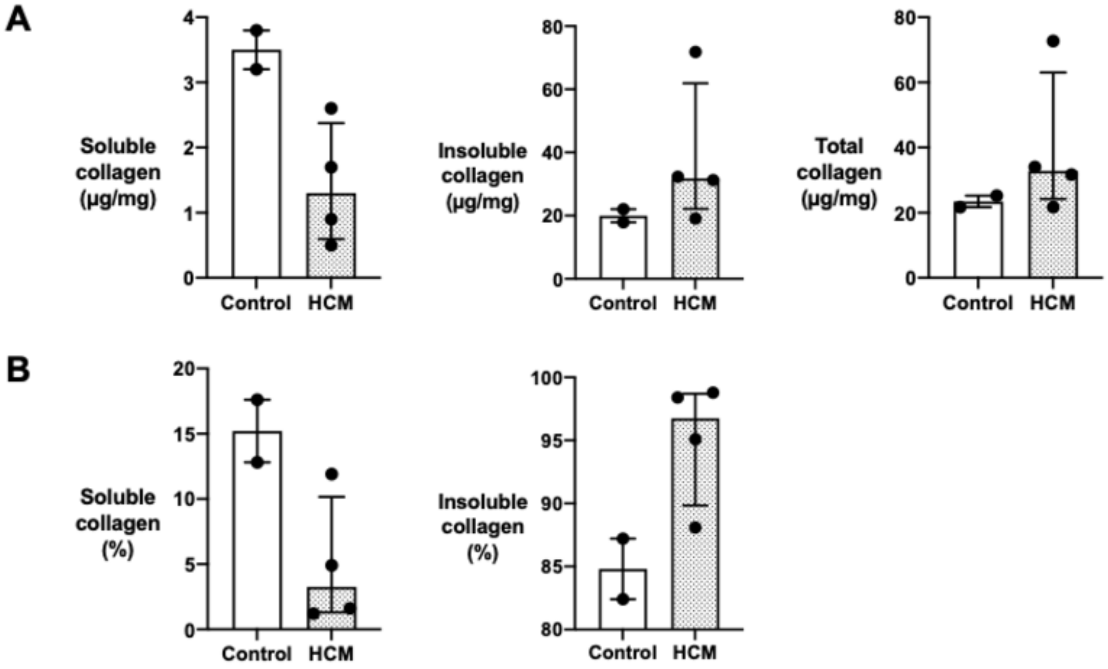

Figure S3. Exploration of soluble and insoluble collagen

(A) Graph showing the measured soluble collagen, insoluble collagen, and total collagen (sum of soluble and insoluble collagen) (µg) in the myocardial samples. (B) Graph showing the fraction of soluble and insoluble collagen (%) in the myocardial samples.

Figure S4. Comparison of immunostaining of LOX on kidney tissues from cat and mouse

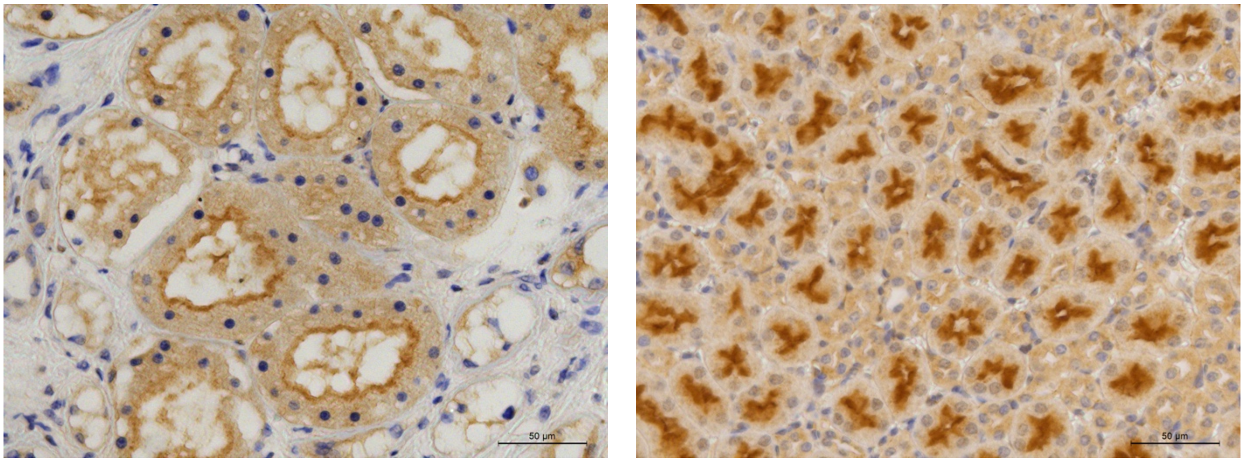

Cat Mouse

Figure S4. Comparison of immunostaining of LOX on kidney tissues from cat and mouse

Kidney is known to express LOX and it is used as a positive control. The antibody is confirmed to show reactivity in mouse by the manufacturer. The similar immunostaining pattern between the cat and mouse suggests the cross-reactivity of the antibody on feline tissue.
