# Supplementary material for: Exploration of Mediators Associated with Myocardial Remodelling in Feline Hypertrophic Cardiomyopathy
Source: Animals (Basel). 2023 Jun 26;13(13):2112. doi: 10.3390/ani13132112 (PMC10339868; doi:10.3390/ani13132112)
Supplement: Supplementary file 1 [file animals-13-02112-s001.zip › Table S1.pdf]

Table S1. Signalment, echocardiographic parameters, and histopathology diagnosis.

Identifier	Breed	Gender	Age (yrs)	Weight (Kg)	BCS	La/Ao	La minor (mm)	IVSd (mm)	LVFWd (mm)	LV FS%	SAM	SEC yes/no	LA Thrombus yes/no	RVFW (mm)	IVS (mm)	LVFW (mm)	Histopath-ology
**Control 1**	Maine coon	MN	9.5	5.8	-	1.5	15.9	4.6	4.8	57	No	No	No	-	-	-	Normal
**Control 2**	DSH	MN	11.3	5.5	-	1.4	-	-	-	-	-	No	No	-	-	-	Normal
**Control 3**	Russian Blue	FS	14	-	-	1.35	13.8	4.7	4.7	57	No	No	No	4.0	7.0	6.5	Normal
**Control 4**	DSH	MN	1.6	4.9	-	1.33	-	-	-	-	-	-	No	-	-	-	Normal
**Control 5**	Russian blue	FS	16	-	-	1.52	14.2	5.0	4.8	60	No	No	No	4.0	7.0	5.5	Normal
**Control 6**	DSH	F	<3	-	5/9	1.41	-	-	-	-	-	No	No	2.0	5.5	6.0	Normal
**Control 7**	DSH	F	<3	-	5/9	-	-	-	-	-	-	No	No	3.0	5.0	5.5	Normal
**Control 8**	DSH	MN	5.5	4.4	5/9	1.35	-	-	-	-	-	No	No	2.0	6.5	7.0	Normal
**Control 9**	DSH	F	5.8		5/9	1.27	-	-	-	-	-	No	No	1.0	4.0	5.5	Normal
**Control 10**	DSH	F	<3	-	5/9	-	-	-	-	-	-	No	No	2.5	6.0	5.5	Normal
**HCM 1**	BSH	MN	13	4.2	4/9	1.56	17	6.5	6.8	65.4	No	No	No	4.0	8.5	10.0	HCM
**HCM 2**	DSH	FS	8.9	4.1	5/9	1.61	18	6.2	6.4	-	Yes	No	No	2.5	8.0	8.0	HCM
**HCM 3**	DSH	MN	8.4	6.2	-	1.82	20.6	8.5	6.9	28.5	Yes	No	No	4.0	12.0	10.0	HCM
**HCM 4**	Maine coon	MN	10.9	5.0	4/9	1.9	25.4	6.2	6.1	49	No	No	No	-	-	-	HCM
**HCM 5**	BSH	MN	6.9	-	-	2.28	26	7.5	8.4	61	No	Yes	No	-	-	-	HCM
**HCM 6**	Bengal	FS	15	3.1	-	1.63	17	7.3	6.5	40.2	Yes	Yes	No	3.0	10.0	8.0	HCM
**HCM 7**	DSH	MN	7	-	-	1.7	-	-	-	-	-	No	No	3.0	10.0	9.5	HCM
**HCM 8**	Tonkinese	FS	9	4.4	-	2.26	16.9	7.3	7.1	61	No	No	No	2.0	8.0	7.5	HCM
**HCM 9**	DSH	FS	1.7	3.0	-	2.5	-	-	-	-	-	No	No	2.5	7.0	7.0	HCM
**HCM 10**	DSH	MN	5	4.2	-	2.6	-	-	-	-	-	Yes	No	-	-	-	HCM

HCM, hypertrophic cardiomyopathy; DSH, domestic shorthair; MN, male neutered; FS, female spayed; BCS, body condition score; La, left atrium; Ao, aorta; IVSd, thickness of interventricular septum in diastole; LVFWd, thickness of left ventricular freewall in diastole; LV FS%, left ventricular fraction shortening (%); SAM, systolic anterior motion; SEC, spontaneous echo contrast; RVFW, thickness of right ventricular freewall of fixed heart; IVS, thickness of interventricular septum of fixed heart; LVFW, thickness of left ventricular freewall of fixed heart
