# Supplementary material for: Exploration of Mediators Associated with Myocardial Remodelling in Feline Hypertrophic Cardiomyopathy
Source: Animals (Basel). 2023 Jun 26;13(13):2112. doi: 10.3390/ani13132112 (PMC10339868; doi:10.3390/ani13132112)
Supplement: Supplementary file 1 [file animals-13-02112-s001.zip › Table S2.pdf]

Table S2. Clinical presentation and imaging summary.

Identifier	Presenting Signs	Murmur	Arrhythmia	Gallop	Co-morbidities	CHF	Reason for euthanasia	Echocardiography summary	Cardiac POCUS
**Control 1**	Coughing	Yes	No	No	Solitary pulmonary carcinoma lesion	No	Owner request	Normal	-
**Control 2**	Lethargy	No	No	No	Hepatic carcinoma	No	Owner request	-	Normal
**Control 3**	Lethargy, weight loss	No	No	No	IRIS II CKD (normal SBP), cognitive dysfunction	No	Cognitive dysfunction	Normal	-
**Control 4**	Trauma	No	No	No	Road trauma	No	Fractured pelvis and urethral rupture	-	Normal
**Control 5**	Lethargy, weight loss	No	No	No	Pancreatic neoplasia	No	Owner Request	Normal	-
**Control 6**	Normal	No	No	No	None	No	Unsociable behaviour	-	Normal
**Control 7**	Normal	No	No	No	None	No	Aggression soiling in house	-	Normal
**Control 8**	Anuria	No	No	No	Toxicity/Acute Kidney Injury	No	Progressive hyperkalaemia	-	Normal
**Control 9**	Road Trauma	No	No	No	None	No	Multiple trauma	-	Normal
**Control 10**	Normal	No	No	No	None	No	Unsociable behaviour	-	Normal
**HCM 1**	Lethargy, shallow breathing	No	No	Yes	None	Yes	Financial constraints	Mild LAE, LVH, pleural fluid	-
**HCM 2**	Anorexia weight loss murmur	Yes	No	no	Hepatic/pancreatic disease "Triaditis"	No	Poor prognosis	Mild LAE, LVH	-
**HCM 3**	Hypotension, hypothermia	Yes	No	No	None	Yes	Poor prognosis	LAE, Severe LVH, Pleural fluid	-
**HCM 4**	Lethargy, inappetence	No	No	No	None	Yes	Poor prognosis	LAE, LVH<br>Pleural fluid	-
**HCM 5**	Hind limb paresis and pain	No	No	No	None	Yes	ATE, poor prognosis	LAE, severe LVH, minimal pleural fluid	-
**HCM 6**	Hind limb paresis and pain	Yes	Yes	Yes	None	No	ATE, poor prognosis	LAE, LVH	-
**HCM 7**	Tachypnoea, inappetence	Yes	No	Yes	None	Yes	Financial constraints	-	LAE, LVH, pleural fluid
**HCM 8**	Tachypnoea, stupor, hypotension	No	Yes	No	Azotaemia due to diuresis	Yes	Poor prognosis	LAE, LVH, moderate pleural effusion, pulmonary oedema	-
**HCM 9**	Collapse episode	Yes	Yes	No	None	No	Owner request	-	LAE, LVH
**HCM 10**	Hind limb paresis and pain	No	Yes	No	None	No	ATE, died in clinic	-	LAE, SEC, LVH

HCM, hypertrophic cardiomyopathy; IRIS, international renal interest society; CKD, chronic kidney disease; SBP, systolic blood pressure; CHF, congestive heart failure; POCUS, point-of-care ultrasound; ATE, aortic thromboembolism; LAE, Left atrial enlargement; LVH, Left ventricular hypertrophy; SEC, spontaneous echo contrast
