# Supplementary material for: Exploration of Mediators Associated with Myocardial Remodelling in Feline Hypertrophic Cardiomyopathy
Source: Animals (Basel). 2023 Jun 26;13(13):2112. doi: 10.3390/ani13132112 (PMC10339868; doi:10.3390/ani13132112)
Supplement: Supplementary file 1 [file animals-13-02112-s001.zip › Table S3.pdf]

Table S3. Information on echocardiography.

	LA/Ao	LVFWd and IVSd	FS (%)
View	RPSAX at the level of the aortic valve	RPLAX4ch, RPLAX5ch, and RPSAX at the level of the papillary muscles	-
Imaging modality	2D Imaging	2D Imaging	-
Timing	Beginning of diastole, the first frame of aortic valve closure	End-diastole, the last frame before the aortic valve opens (RPLAX5ch), the first frame after the mitral valve closes (RPLAX4ch), or when the left ventricular internal diameter was the largest (RPSAX)	-
Measurement	Ao: From the blood-tissue interface at the midpoint of the right aortic sinus to the commissure between the noncoronary and left coronary aortic cusps<br>LA: Extension of the aortic line to the blood-tissue interface of the left atrial wall, immediately lateral to the pulmonary vein	Leading edge technique avoiding the papillary muscles or false tendon attachments<br>Average of 3 measurements from the area that measures the maximal thickness is used	Calculated using (LVIDd−LVIDs)/LVIDd

Abbreviations: RPSAX, right parasternal short axis view; RPLAX4ch, right parasternal long axis 4 chamber view; RPLAX5ch: right parasternal long axis 5 chamber view; LVIDd and LVIDs: Left ventricular internal diameter end diastole and end systole; LA: left atrium; Ao: aorta; FS: fraction shortening
