# Supplementary material for: Exploration of Mediators Associated with Myocardial Remodelling in Feline Hypertrophic Cardiomyopathy
Source: Animals (Basel). 2023 Jun 26;13(13):2112. doi: 10.3390/ani13132112 (PMC10339868; doi:10.3390/ani13132112)
Supplement: Supplementary file 1 [file animals-13-02112-s001.zip › Table S4.pdf]

Table S4. Sequence and source of primers.

Gene	Primer sequence<br>Forward (5' → 3')<br>Reverse (5' → 3')	Amplicon size (bp)	Ensembl Transcript Number / Accession Number
TGFB1	GGAATGGCTGTCCTTTGATG<br>TGCAGTGTGTTATCTTTGCTGTC	120	AY425617
TGFB2	CTTGTGTTCCAGACATTCC<br>ACGTCAAATCGGACAATTC	96	XM_003999507
ACTA2	ACGGTTCTGGGCTCTGTAAGG<br>GTGGTGCCATATCTTTTCCATATCGT	230	XM_003993871.5
LUM	ACTCCCCAAGTCCCTGGT<br>GAGCCAAGCTTCGTTATCTTGT	60	ENSFCAT00000003763
LOX	ACAAAGCGAGTTTCTGTCTTGAGG<br>GTTCACGCTGACCTTTAGAATGTAGT	188	XM_023254614.1
LOXL2	GCCCATCTGGTTGGACAATATCTAC<br>CTGGATGTTCAGGTTCTCTATGTGG	190	XM_003984692.4
COL1A1	AGGTCCTTCCGGAGCTTCT<br>ACCGTTGAGTCCGTCCTTC	85	ENSFCAT00000004567
COL3A1	CTGGCCAGCCTGGAGATA<br>CCATTCTGTCCAGGAGCAC	139	ENSFCAT00000014744
RPS7	GTCCCAGAAGCCGCACTTTGAC<br>CTCTTGCCCACAATCTCGCTCG	81	AY800278
RPL30	CCTCGGCAGATAAATTGGACTGTC<br>TGATGGCCCTCTGGA ATTTGAC	111	AY700577
